# Antimicrobial action of an endophytic fungi from *Sophor flavescens* and structure identification of its active constituent

**DOI:** 10.1080/13102818.2014.911618

**Published:** 2014-07-15

**Authors:** Na Yu, Lu He, Na Liu, Yong Wang, Hongbo Xu, Dandan Liu

**Affiliations:** ^a^ School of Chemical Engineering, University of Science and Technology Liaoning,Anshan City, Liaoning Province, People's Republic of China; ^b^ Shanghai Key Lab of Protected Horticultural Technology, Shanghai Academy of Agricultural Sciences, Horticultural Research Institute,Shanghai City, China People's Republic of China

**Keywords:** *Sophora flavescens*, endophytic fungi, antimicrobial substance, structure analysis, isolation, TLC biological autoradiography

## Abstract

Endophytic fungus BS002 was isolated and characterized from *Sophora flavescens* by plate method, which has broad antimicrobial activity. Isolation and trace of a new bioactive compound from the fungus’ culture extracts with the method of column chromatography and TLC biological autoradiography was conducted. Finally, it was identified as 6,7-(2′E) dibutenyl-5,8-dihydroxy-(Z)-cyclooct-2-ene-1,4-dione by nuclear magnetic resonance, infrared and liquid chromatography–mass spectrometry. The compound presented strong antifungal activities for example: *Botryosphaeria berengriana* f.sp.* piricola, Physalospora piricola, Cladosporium cucumerinum *Ell*. Arthur., Fusarium oxysporum f.sp. cucumerinum, Fusarium moniliforme.* The inhibition to *Physalospora piricola* was the strongest with an antibacterial diameter of 45 mm. This paper is the first report of the antimicrobial activity of endophytic fungi BS002 that was the secondary metabolites extracted from the seeds of *Sophora flavescens*. The results provide a broad foreground for biopharmaceuticals and biopesticide.

## Introduction

Endophyte is an endosymbiont, which lives within a plant for at least part of its life without causing apparent disease.[1] On earth, some endophytes are widely distributed, easily mutated, and their secondary metabolites have a variety of biological activities. Endophytic fungi have roles to produce plant growth regulators, promote the growth and enhance the stress resistance of host plants.[2–4] Endophytic fungi living in the healthy tissues or organs of plants are potential sources of new natural products for exploitation in medicine, agriculture and industry.[5,6] Today, the medicinal resources of animals and plants are dwindling. Thus, the research and development of endophytes have far-reaching prospect and value. Over the past decades, a new group of antibiotics with antimicrobial, insecticidal and anticancer characteristics have been isolated from endophytic fungi.[7–13] Caruso screened 150 kinds of endophytic fungi from *Taxus mairei* which had good antitumor effects.[14,15] Martha reported that bioactive compounds against *Phytophthora capsici* was isolated from a newly discovered endophytic fungi.[16]

*Sophora flavescens* (*Sophora flavescens* Ait), a medicinal plant, has medicinal functions such as antimicrobial, insecticidal, anticancer, antiviral and others. It is hardly infected with plant diseases and insect pests during its life and has been used as Chinese traditional medicine for more than 2000 years. The main chemical components of *S. flavescens* are alkaloid and flavonoid compounds,[17–19] which have been evaluated for many medicinal effects.[20–22] Liu et al. proved that matrine have the effect of anti-endotoxin [23] and can destroy endotoxin molecules. Ji-Sang et al. Proved that formononetin.1 isolated from *S. flavescens* can cause the inhibition to the activity of monoamine oxidase.[24,25] At present, more and more studies focus on the endophytic fungi extracted from various medicinal plants for antitumour activity, however, there are few reports concerning isolation and identification of the endophytic fungi from *S. flavescens* and researching their secondary metabolites. In our previous work, we obtained an endophytic fungus (strain number BS002) showing great antioxidant activity from the seeds of healthy *S. flavescens*. As part of our on-going search for bioactive substances from microorganisms, it was found that the fermented broth of BS002 showed broad antimicrobial activity. In this paper, the antimicrobial activity of the endophytic fungus BS002 and identification of its antimicrobial component were described, which have never been reported, to the best of our knowledge. The study was completed in January 2013.

## Materials and methods

### Endophyte separation

Healthy *S. flavescens* were collected in Xiangya Mountain, Kaiyuan and Liaoning Provinces in China. The endophytes were isolated from the seeds of *S. flavescens* according to the plate method [26] as follows: the seeds were soaked with 70% (v/v) ethanol for 1 min and 0.1% (v/v) HgCl_2_ for 3 min, and three times washed with sterile water; then cut into 0.5 cm × 0.5 cm small pieces to be put on fresh potato dextrose agar (PDA) plates (six on each plate) containing streptomycin (50 U/mL), and they were incubated on plates at 25 °C. The growing mycelia were transferred and purified on PDA by picking mycelium tip, repeatedly purified until a pure strain was obtained. After surface sterilization, samples were not cut to culture epiphytes. They were observed for colonies under the same conditions and if not, it showed that the sterile processing of flavescens surface was complete. The isolated endophytic fungi BS002 was stored on PDA slants at 4 °C and kept at College of Biological Engineering, University of Science and Technology LiaoNing, Liaoning Province, China.

### Antimicrobial assay

Antimicrobial activity was determined by the agar-well diffusion method.[27] The tested microorganisms were provided by Biological Engineering, University of Science and Technology LiaoNing (Liaoning, China), including 16 pathogenic bacteria (*Herba Pogostemonis*, *Staphyloccocus aureus*, *Bacillus subtilis* Cohn, *Erwinia carotovora* subsp., *Pseudomonas aeruginosa*, *Bacillus thuringiensis*, *Bacillus mucilaginosus Krassilnikov*, *Bacillus subtilis*, *Ralstoia solanacearum* Smith, *Bacillus sphaerieus*, *Pondushydrogenii*, *Bacillus cereus*.Frankland, *Enterobacter aerogenes*, *Proteusbacillus vulgaris*, *Azotobacter chroococcum Beijerinck*, *Escherichia coli*) and 24 pathogenic fungi (*Physalospora piricola*, *Colletotrichum gloesporioides*, *Sclerotinia sclerotiorum*, *Gibberella sanbinetti*, *Botrtyis cinerea* Pers, *Cytospora* sp., *Fulvia fulva Ciferri*, *Thanate phorus sasakii* lto., *Phytophthora capsici*, *Colletotrichum orbiculare* Arx, *Cladosporium cucumerinum* Ell. Arthur., *Colletotrichum coccodes* Hughes., *Coniothyrium diplodiella* Sacc., *Verticillium dahliae*, *Alternaria mali* Roberts, *Bipolaris sorokiniana*, *Botryosphaeria berengriana* f.sp. piricola, *Cercospora musae* Zimm, *Fusarium* sp., *Fusarium oxysporum* f.sp. cucumerinum, *Ustilaginoidea virens* Tak., *Fusarium moniliforme*, *Rhizoctonia cerealis*, *pyricutaria oryzae* Cav.). First, the suspension of pathogenic fungi or bacteria should be prepared, and mixed with PDA medium or beef extract-peptone medium, respectively, to be poured in plates. One endophytic fungi block was placed in the centre of a plate. They were incubated in plates at 25 °C, and the sizes of the inhibition zones were measured after 72 h.

### Identification of endophytic fungus BS002

Insert method [28] was used for morphological identification of endophytic fungus BS002. Coverslip was inserted into the medium with a 45° angle, the depth was approximately 1/3 of the coverslip, it was upside-down cultured at 25 °C and observed by a microscope.

For microscopic examination, endophytic fungus BS002 was grown at 25 °C on potato sugar agar (PSA) for 48–72 h in dark conditions. The edge of the mycelial colony was cut into small pieces of 2–3 mm, and four to five of them were moved to czapek culture broth at 26 °C, 120 rpm for four days. The dried mycelium was collected, and was ground into powder for further use. The method and reagents for DNA extraction were performed according to a modified protocol of Zhu et al.[29]

White et al. [30] reported that the used primers for ITS amplification were ITS1 (5′-TCC GTA GGT GAA CCT GCG G-3′) and ITS4 (5′-TCC TCC GCT TAT TGA TAT GC-3′) (Biological Engineering Services Co., Ltd.), The thermal cycling program for polymerase chain reaction (PCR) were as follows: 3 min of initial denaturation at 94 °C followed by 30 cycles of denaturation at 94 °C for 1 min, primer annealing at 55 °C for 30 sec, extension at 72 °C for 1 min and a final extension at 72 °C for 20 min. The sequencing work was commissioned to Geokon Biotechnology Co., Ltd. The obtained DNA sequence was submitted to GenBank for homology analysis by BLASTN program.

### Isolation and purification of the active compounds from cultures in shaken flasks

For initial screening, endophytic fungi BS002 was inoculated in PD medium (potato 200 g, sugar 10 g, glucose 10 g, sodium acetate 1.66 g, peptone 1.02 g, water 1000 mL) at 25 °C, 150 rpm training for three days, and then filtrated. The culture fluid was separated from the mycelia by filtering. The filtrate was evaporated and concentrated to 200 mL at 40 °C, and was ready for further use.

The concentrate was extracted by ethyl acetate, and then mixed with HPD-722 macroporous resin, static adsorbed for 40 min, and filtrated. Then, 0.3 L wet resin was put into Φ4 cm × 55 cm glass column, eluted with concentration gradient of 80%, 60%, 40%, 20% and 10%. The separated production was tracked by the method of TLC autoradiography.[24] The developing agent was chloroform:methanol in a ratio 5.5:1.5. The plates were cultured at 25 °C for observation of the antibacterial effect. Then the fraction with antibacterial activity was collected, and concentrated to 1–2 mL for vacuum freeze-drying.

### Chemical characterization of the active compound

The substance with antibacterial activity was analysed by liquid chromatography–mass spectrometry (LC-MS) (1100 LC-MS APCI mass spectrometer, provided by Agilent). The Sim-pack was C18 Agilent; the mobile phase was methanol:water (80:20); the added quantity was 20 μL; the column temperature was 25 °C; the wavelength for detection was 254 nm. Then the peak was identified by MS, and the molecular weight was determined. 1H-NMR (500 MHz), 13C-NMR (125 MHz), H1-H1-COSY, DEPT-135 and HSQC spectrum (AV 500 nuclear magnetic resonance (NMR) instrument, Bruker Company) were used to analyse the structure of the unknown compound with solvent of MeOD, then, the structure was validated by MS.

## Results and discussion

In this paper, the compound inhibited greatly the growth of *Botryosphaeria berengriana* f.sp. *piricola, Physalospora piricola, Cladosporium cucumerinum *Ell*. Arthur., Fusarium oxysporum* f.sp. *cucumerinum, Fusarium moniliforme*. The inhibition of *Physalospora piricola* was strongest with an antibacterial diameter of 45 mm. In order to determine the endophytic fungus BS002, molecular techniques were conducted, the fruiting bodies of endophytic fungus BS002 were observed with a typical structure of the aerial mycelium of the fungi, etc. According to the analysis of ITS and 18S rDNA sequences, endophytic fungus BS002 can be identified as *Penicillium* sp. M-01 (a variant of *Penicillium* sp.).

### Screening and identification of the endophyte BS002 with broad-spectrum antimicrobial activity

Five strains of endophytic fungi were isolated from healthy seeds of *S. flavescens*. Screening of the antimicrobial activity of all strains was conducted. All 5 strains exhibited significant antimicrobial activity against several agricultural pathogenic fungi, and the culture filtrate from endophytic fungus BS002 showed the best antimicrobial activity, especially against *Physalospora piricola*.

**Figure 1.**
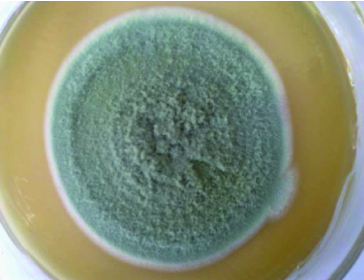
Colony of endophytic fungus BS002.

**Figure 2.**
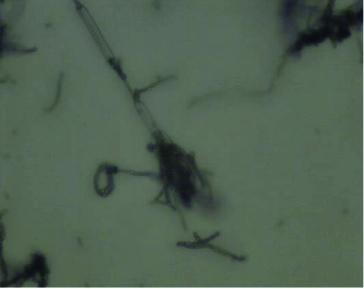
Fruiting bodies morphology of endophytic fungus BS002 (400×).

On PDA medium, the colony of fungus BS002 was dark green, short villous and had regular white margins (Figure 1). Mycelium was coarser, intertwined, transparent and phrenic. Mature strains had fruiting bodies that were the typical structures of the aerial mycelium of the fungi. The spores were on broom-like branches, and they were round or oval (Figure 2).

A part from the ITS sequences of the BS002 strain was amplified by PCR with the universal primers ITS1/ITS4, and about 500 bp sequences of rDNA fragments were obtained; these were analysed and compared to the sequences in GenBank database using the BLAST program. The results showed a similarity of the ITS sequences of the endophytic fungus BS002 and to the ITS sequences of *Penicillium* sp. M-01 reached 100%. On the basis of its ITS and 16s rDNA sequences, the BS002 strain was *Penicillium* sp. M-01, and it was a variant of *Penicillium* sp.

### Antimicrobial activity of endophytic fungus BS002

Endophytic fungus BS002 was assayed for its antimicrobial activity against the tested 16 pathogenic bacteria and 24 pathogenic fungi, as shown in Table 1. The strain did not inhibit the growth of the 16 bacteria, but the endophytic fungus BS002 exhibited strong inhibitory activities against the tested pathogenic fungi including *Botryosphaeria berengriana* f.sp. piricola, *Physalospora piricola*, *Cladosporium cucumerinum* Ell. Arthur., *Fusarium oxysporum* f.sp. cucumerinum, *Fusarium moniliforme*, etc. Compared to the controls, the endophytic fungus BS002 had a broad-spectrum antimicrobial activity. The result could indicate that the extract from the endophytic fungus BS002 may be used as antibiotic in agriculture.

**Table 1.** Antimicrobial activity of the endophytic fungus BS002.

Pathogenic fungi	Antibacterial diameter (mm)	Pathogenic fungi	Antibacterial diameter (mm)
*Phytophthora capsici*	14 aA	*Cladosporium cucumerinum* Ell. Arthur.	21 aA
*pyricutaria oryzae* Cav.	8 aA	*Rhizoctonia cerealis*	13 aA
*Fusarium moniliforme*	13 aA	*Ustilaginoidea virens* Tak.	8 aA
*Fusarium oxysporum* f.sp. *cucume-rinum*	17 aA	*Fusarium* sp.	12 aA
*Cercospora musae Zimm*	10 aA	*Botryosphaeria berengriana* f.sp. *piricola*	27 aA
*Colletotrichum orbiculare Arx*	14 aA	*Colletotrichum coccodes* Hughes.	8 aA
*Colletotrichum gloesporioides*	7 aA	*Coniothyrium diplodiella* Sacc.	11 aA
*Sclerotinia sclerotiorum*	9 aA	*Verticillium dahliae*	10 aA
*Gibberella sanbinetti*	8 aA	*Alternaria mali* Roberts	12 aA
*Physalospora piricola*	45 aA	*Thanate phorus sasakii* lto.	7 aA
*Botrtyis cinerea* Pers	8 aA	*Bipolaris sorokiniana*	8 aA
*Cytospora* sp.	9 aA	*Fulvia fulva Ciferri*	13 aA

Note: Different small and capital letters mean significant differences at 0.05 and 0.01 levels, respectively.

### Structure elucidation of antimicrobial compound

**Figure 3.**
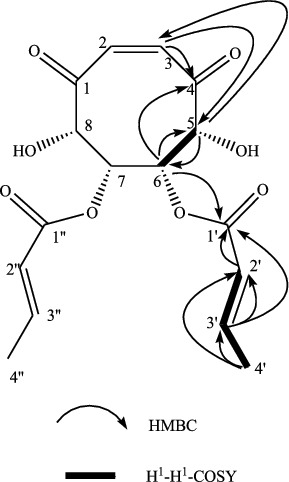
The H^1^-H^1^-COSY and the key HMBC of the new compound.

The compound with strong antimicrobial activity was identified as 6,7-(2′E)dibutenyl-5,8-dihydroxy-(Z)-cyclooct-2-ene-1,4-dione on the basis of the experimental results obtained in our laboratory. Its chemical structure was shown at Figure 3. This compound was white powder (methanol), mp 183 °C, [α]_D_ +3.6°. The ion peak was *m*/*z* 339.1072[M+H]^+^ (calcd. 339.1080), given by HR-TOF-MS, and its molecular formula (C_16_H_18_O_8_) was determined. The infrared spectrum gave strong wide peak in ν_OH_ 3383 cm^−1^, a conjugate strong absorption peak ν_C=O_ in 1695 cm^−1^, and an absorption peak ν_C=C_ in 1633 cm^−1^. Ultraviolet spectrum λ_Max_ 309 nm (*ϵ* 3.20), and combined with its NMR, it could be speculated that there was a conjugated system containing unsaturated carbonyl in the structure of the molecule. The following was documented ^1^H-NMR (500 MHz, MeOD): δ6.81 (1H, dq, *J* = 15.9, 6.6 Hz), δ6.43 (1H, d, *J* = 15.9 Hz), δ6.09 (1H, s); δ4.23 (1H, br s), δ4.82 (1H, br s); δ1.90 (3H, d, *J* = 6.6 Hz); δ6.81 (1H, dq, *J* = 15.9, 6.6 Hz), δ6.43 (1H, d, *J* = 15.9 Hz), δ1.90 (3H, d, *J* = 6.6 Hz). Also ^13^C-NMR (125 MHz, MeOD) δ205.9 (C=O), δ170.9 (C=O); three carbon signals, δ143.5 (C=C), δ124.6 (C=C), δ124.5 (C=C); δ80.5 (C–O), δ76.1 (C–O); δ18.9 (–CH_3_). According to the combined data of NMR and mass spectrum it could be speculated that the compound is a symmetric structure.

The structure of this compound was deduced by two-dimensional NMR technology, such as H^1^-H^1^-COSY, DEPT, HSQC and HMBC (Figure 3). One of the important HMBC remote related signal was: H-3 (δ_H_ 6.09) and C-4 (δ_C_ 205.9), C-5(δ_C_ 80.5); H-5 (δ_H_ 4.23) and C-3 (δ_C_ 124.6), C-6 (δ_C_ 76.1); H-6 (δ_H_ 4.82) and C-4 (δ_C_ 205.9), C-5 (δ_C_ 80.5), C-1′ (δ_C_ 170.9); H-2′ (δ_H_ 6.43) and C-1′ (δ_C_ 170.9); H-3′ (δ_H_ 6.81) and C-1′ (δ_C_ 170.9), C-2′ (δ_C_ 124.5); H-4′ (δ_H_ 1.90) and C-2′ (δ_C_ 124.5), C-3′ (δ_C_ 143.5). According to the wide singlet type of H-5 and H-6, it could be judged that H-5 and H-6 are in the same plane. This compound is the derivative of cyclooct-2-ene-ketone,[21] and named 6,7-(2′E) dibutenyl-5,8-dihydroxy-(Z)-cyclooct-2-ene-1,4-dione (Table 2).

**Table 2.** Data of the ^13^C-NMR (125 MHz) and ^1^H-NMR (500 MHz) (MeOD, δ in ppm).

Position	C	H
1	205.9	
2	124.6	6.09(1H, s)
3	124.6	6.09(1H, s)
4	205.9	
5	80.5	4.23(1H, br s)
6	76.1	4.82(1H, br s)
7	76.1	4.82(1H, br s)
8	80.5	4.23(1H, br s)
1′	170.9	
2′	124.5	6.43(1H, d)
3′	143.5	6.81(1H, dq, *J* = 15.9, 6.6 Hz)
4′	18.9	1.90(3H, d, *J* = 6.6 Hz)
1″	170.9	
2″	124.5	6.81(1H, dq, *J* = 15.9, 6.6 Hz)
3″	143.5	6.81(1H, m)
4″	18.9	1.90(3H, d, *J* = 6.6 Hz)

Note: All spectra were recorded on AV500, in MeOD, δ in ppm.

### Final remarks

We expected to obtain some structures which were the same or similar with the active composition from *S. flavescens* by studing the secondary metabolites of the endophytic fungus BS002. However, the obtained substance with antimicrobial activity was different from the known compounds. It had a cyclic symmetric olefin structure with unsaturated double bonds and carbonyl group, and lively physical and chemical properties. At temperatures greater than 55 °C or under slightly alkaline conditions the compound easily lost its activity in the test. We believe that this compound with a broad-spectrum antifungal activity may be put to development as a new biopharmaceutical product.

## Conclusions

In this research, endophytic fungus BS002 (*Penicillium* sp. M-01, a variant of *Penicillium* sp.) was isolated from the seeds of healthy *S. flavescens* for the first time, and the antibacterial substance was identified as 6,7-(2′E) dibutenyl-5,8-dihydroxy-(Z)-cyclooct-2-ene-1,4-dione. In this study, we obtained the compound from culture filtrates of BS002. The result provided a green process and a new way to produce this compound. Antimicrobial assay showed that endophytic fungus BS002 inhibited the growth of the tested 24 microorganisms, having a wider antimicrobial spectrum than the positive references. These results suggest that endophytic fungus BS002 has a potent antimicrobial activity and could be a valuable candidate for the discovery of new antimicrobial drugs.
